# Enhancing cardiotocography classification via ensemble learning and threshold optimization

**DOI:** 10.1038/s41598-025-18990-z

**Published:** 2025-11-04

**Authors:** Lingping Kong, Václav Snášel, Zhonghai Bai, Dominik Vilimek, Seyedali Mirjalili, Jeng-Shyang Pan, Jitka Horakova, Radek Martinek, Radana Vilimkova Kahankova

**Affiliations:** 1 Faculty of Electrical Engineering and Computer Science, VŠB-Technical University of Ostrava, Ostrava, Czech Republic; 2 Faculty of Electrical Engineering and Computer Science, Department of Cybernetics and Biomedical Engineering, VŠB-Technical University of Ostrava, Ostrava, Czech Republic; 3 Centre for Artificial Intelligence Research and Optimization, Torrens University Australia, Adelaide, Australia; 4 School of Artificial Intelligence, Nanjing University of Information Science and Technology, Nanjing, China; 5 Obstetrics and Gynecology Department, University Hospital Ostrava, Ostrava, 70080 Czech Republic; 6 University Research and Innovation Center, Obuda University, 1034 Budapest, Hungary; 7 Center for Advanced Technologies and Engineering, CATEN, Ostrava, Czech Republic

**Keywords:** Cardiotocograph, Hypoxemia, Ensemble classifier, Moving threshold, Probalistic random forest, UnderSampling dataset, Computational biology and bioinformatics, Cardiology

## Abstract

Machine learning classifiers trained on imbalanced healthcare datasets often exhibit bias, leading to poor performance on critical cases. The cardiotocography (CTG) dataset exemplifies this issue, where misclassification of pathological cases arises due to both class imbalance and non-optimal probability thresholds. Statistical analysis suggests refining classification thresholds, but this approach has been largely overlooked in CTG data research. To address these challenges, we propose a multifusion method integrating undersampling, threshold-moving optimization, and ensemble classifiers to enhance classification precision while maintaining computational efficiency. Applied to a CTG dataset of 502 cases from Czech Technical University and University Hospital Brno, our method showed significant improvements in identifying pathological cases. While baseline models correctly classified only about 2 out of 11 cases per test, our approach achieved 76.92, 75, and 41.67% precision, accurately identifying 9, 9, and 3 cases out of 12, respectively.

## Introduction

Fetal surveillance^1^ during labor serves the critical purpose of quickly detecting potentially acidotic fetuses^2^ while minimizing unnecessary interventions. In the course of labor, fetuses can experience recurrent episodes of decreased oxygen supply, a natural occurrence, but those with compromised defense mechanisms are susceptible to developing metabolic acidosis^3^. This condition can result in long-term consequences such as neurodevelopmental disabilities, cerebral palsy, or, in severe cases, fetal death. Therefore, continuous fetal monitoring during labor is indispensable, typically performed using a cardiotocograph (CTG)^4^, which simultaneously records fetal heart rate (FHR) and uterine contraction (UC) signals. However, the current CTG assessment is based heavily on visual analysis of various FHR signal patterns, following medical guidelines. Regrettably, this method exhibits significant discrepancies among observers^5^, lacks objectivity, and demonstrates poor interpretive consistency.

To address these challenges, numerous digital assistance systems have been developed to help clinicians interpret CTG results^6^. However, there is insufficient evidence to suggest that these systems effectively reduce the incidence of newborn acidosis without increasing obstetric interventions compared to traditional CTG analysis. Consequently, numerous signal-processing techniques have been explored to uncover concealed FHR characteristics that could distinguish between normal fetal status and acidosis. However, the outcomes obtained so far have not been satisfactory for integration into clinical practice.

Recent clinical research emphasizes the importance of accurately identifying hypoxemia^7^, which requires a thorough understanding of the fetal compensatory mechanisms. These mechanisms, influenced by the autonomic nervous system, adapt the fetus to increased activity levels during perceived oxygen deficiency. The dynamic nature of fetal heart rate (FHR), reflecting the modulation of the autonomic nervous system^8^, contains crucial information on fetal well-being. Conventional signal processing methods may not be suitable for precise CTG assessment as they fail to integrate these physiological characteristics, particularly the nonlinear and nonstationary aspects of FHR resulting from autonomic nervous system modulation.

Moreover, CTG databases, such as the CTU-UHB data derived from the Czech Technical University (CTU) in Prague and the University Hospital in Brno (UHB), often present unbalanced data, where the data set contains significantly fewer samples of ‘Pathological’ cases compared to the ‘Normal’ class^9^. Learning from unbalanced data^10^ poses a challenge to classification algorithms, and this leads to models with inadequate predictive accuracy, particularly in regard to the minority class. This poses a significant issue as the minority class, e.g., the ‘pathological class, usually holds more significance, making the problem more prone to classification errors in the minority class compared to the majority class.

Several studies^11^ have explored classification tasks using the CTU-UHB dataset, each employing different methods, split criteria, and feature sets, as notable from summary in Table 1. The works differ notably in the stage of labor considered (Stage I, Stage II, or unspecified), the selection and size of the data subsets (often using a binary classification approach), and the thresholds used for dividing normal and pathological cases, primarily based on pH values (ranging from 7.05 to 7.20) or additional clinical indicators (e.g., base deficit, Apgar scores, birth weight). Various signal processing and machine learning methods were applied, including spectral and time-frequency analysis (FFT, Wavelet Transform), feature-based approaches (e.g., EMD, recurrence plots, common spatial patterns), and different classifiers (SVM, kNN, ANN, CNN, and fuzzy logic-based models). Some studies relied solely on feature extraction from the signals, while others transformed signals into two-dimensional representations for deep learning. The number of features used for classification also varied considerably, ranging from direct signal transformations without explicit feature extraction to structured sets of selected features based on clinical relevance or optimization algorithms.

**Table 1:** Summary of recent related studies using pH for label formation of classification task on CTU-UHB dataset, where stage corresponds to the part of the labor used, the size (P-S-N) indicates the number of instances for pathological, suspicious, and normal. FFT, fast fourier transform; SVM, support vector machine; RF, random forests; FC$\epsilon$H; DT, decision tree; PRF, probabilistic random forest; CWT, continuous wavelet transform; kNN, *k*-nearest neighbor.

Study	Stage	Size (P-S-N)	Split criteria	Classess	Methods	features
Zarmehri (2019)^12^	I, II	44/-/508	pH (7.05)	2	FFT	–
Zhao (2019)^13^	N/A	105/-/447	pH (7.15)	2	RP, CNN	–
Zhao (2019)^14^	N/A	105/-/447	pH (7.15)	2	CWT, 2D-CNN, DL	–
Fuentealba (2019)^15^	I	18/-/354	pH (7.05,7.20), BDecf(12)	2	SVM	34
Alsaggaf (2020)^16^	N/A	105/-/447	pH (7.15)	2	SVM, ANN, *k*NN	24
			$$AS_{1,5}$$(7), BDecf(12)			
da Silva (2020)^17^	N/A	40/-/358	pH (7.05, 7.20)	2	SVM	20
Jezewski (2021)^18^	N/A	40/-/358	pH (7.05, 7.20)	2	FC$\epsilon$H	12
			BW(5,10), AS(5,6)			
Zeng (2021)^19^	I	27/-/442	pH (7.05)	2	SVM	27
Comert^20^	N/A	177/-/375	pH(7.20)	2	ANN, kNN, SVM, DT	12
Czabanski (2023)^21^	N/A	105/-/105	multiple pH 7.05–7.20	2	FC$\epsilon$H, fuzzy SVM	12
**Proposed study**	** I, II**	**37/114/351**	**pH (7.05, 7.20)**	**3**	**RF, PRF**	**68**

Significant values are in bold.

In this paper, we propose a fusion method that integrates several techniques to address the imbalanced CTU-UHB data and explore decision making on classification beyond the statistics of the classifier. Specifically, we ensemble random forest (RF)^22^ and probabilistic random forest (PRF)^23^ classifiers to improve robustness and anti-noise property. Then, we apply the probability threshold moving strategy to optimize the decision-making on the classification of ‘Pathological’ and ‘Suspicious’ cases, under an assumption that the recognition of the ‘Pathological’ case is more important than the situation of misclassified ‘Suspicious’ cases.

This work presents a targeted approach to CTG classification, emphasizing the pathological class and proposing advanced techniques for enhancing diagnostic accuracy. The following is a summary of our key contributions and innovations.

- *Targeting the Pathological Class* The pathological class holds critical importance in clinical diagnosis, yet it remains largely overlooked in most of the research literature on CTG classification. To bridge this gap, we propose a specialized model designed to improve the accurate identification of pathological cases.
- *Novel Ensemble Classifier with Decision Bias Adjustment* The pathological cases in CTG data are not just an issue of imbalance; they also relate to shifts in decision probability. We introduce a novel ensemble classifier that leverages widely recognized public classifiers while incorporating a fine-tuned decision bias adjustment strategy. This approach not only enhances classification performance but also demonstrates robustness against noise, making it well-suited for medical imbalance data applications
- *Extensive Sampling Experiment for Medical Data* Furthermore, we conducted extensive experiments on various sampling methods for CTG dataset classification. Although sampling strategies have been explored in different domains, their application to medical data from CTG has remained underexplored with no informative outputs. Our findings confirm that undersampling is particularly effective for medical data, as it mitigates bias and minimizes the influence of unreliable synthetic data, an issue that manifests differently in non-medical datasets.
- *Optimizing Probability Thresholds Using Cohen’s Kappa* A key contribution of our work is in optimizing decision-making probability thresholds, a challenge that conventional classifiers cannot fully address. To achieve this, we employ Cohen’s kappa as an optimization metric, ensuring a more balanced classification of pathological cases, often the minority class yet the most clinically relevant in many scenarios.

The remainder of the paper is organized as follows. As outlined in the Introduction, we revisit the challenges associated with cardiotocography (CTG) classification and emphasize its significance. Subsequently, we provide a concise review of the current state of research, highlighting existing gaps. Our proposed approach to CTG classification experiments and results, including a detailed analysis with comparative insights, is presented in Section Results. In Section Discussion, we thoroughly discuss the findings, interpret the outcomes, and provide valuable insights into the methods and results. Finally, Section Methods introduces the proposed fusion method for CTG data classification, incorporating threshold optimization to enhance decision-making.

## Results

We conducted two experiments. First, we verified the effectiveness of applying threshold tuning. Second, we compared the results of the proposed method with those of the baseline classifier. Specifically, we used 3-Fold Stratified Cross-Validation. Therefore, there are three corresponding classification reports for each experiment.

### Ablation comparison between with and without threshold tuning

Tables 2, 3, 4, and 5 display the comparison results and classification reports between the classification method with and without the threshold moving strategy on three stratified data split tests. The classification report presents the precision, recall and F1 score outcomes for each classification class: ‘Normal’, ‘Suspicious’, and ‘Pathological’. The *support* column also indicates the number of instances in the corresponding class.

In each result table, the left side presents the classification report of the method without the threshold moving optimization. We also include the classification confusion matrix for each table (*CM*), where each column presents the true label, and the row presents the predictions. We denote the confusion matrix of the method without optimization as $CM_{default}$, and $CM_{optimized}$ represents the result with the threshold-moving optimization. The subscript indicates the index of the test data, such as $CM_{default}^1$, which is the confusion matrix from the method without optimization on the first test split data.

From Table 2, we observe that the classification score for ‘Normal’ cases is the same as 1.00, both with and without the optimization method. In the ‘Pathological’ class, the recall increases by 0.15 from 0.62 to 0.77 due to optimization, and the f1-score remains with a slight decrease as the one without the optimization. From the overall performance view, there is significant performance loss in suspicious cases. There is a 0.26 score decrease in recall on ‘Suspicious’ by the optimization problem over the method without threshold moving, as the threshold is designed to prioritize ‘Pathological’ cases over ‘Suspicious’ ones, for which doctors require proper chemical care. Examining the confusion matrices $CM_{default}^{Table\,2}$ and $CM_{optimized}^{Table\,2}$, we observe that the method with threshold optimization tends to classify ‘Pathological’ cases more accurately and reduces the probability of classifying a case as ‘Suspicious’ out of caution. We assign the labels to the signal by the pH value, where the boundary of the pH is a hard threshold yet is sensitive to the result of the actual case. Table 3 shows optimization results on function score ($f_{score}$) and corresponds to the optimized threshold ($\lambda$), refer Line 12 in Alg 1, where $f_{score}$ corresponds to the score related to Cohen-Kappa score and the precision score on Pathological cases. We observe that the $f_{score}$ achieves the lowest value when the $\lambda$ without tuning.

Table 4 presents the classification report for the second test. A similar trend is observed in $CM_{default}^{Table\,4}$ and $CM_{optimized}^{Table\,4}$, where the method with threshold optimization favors accurate classification of ‘Pathological’ cases rather than categorizing them as ‘Suspicious’. Therefore, in Table 4, the method with threshold optimization achieves a higher recall score and a lower precision score for ‘Pathological’ cases. However, using both methods, the classification accuracy remains the same for Normal‘ cases. Table 5 yields similar results as in Tables 2 and 4, with the confusion matrices, as $CM_{default}^{Table\,5}$ and $CM_{optimized}^{Table\,5}$, respectively, favoring the ‘Pathological’ cases correctly classified over the ‘Suspicious’ ones.

$$CM^{Table\,2}_{default}= {\mathop { \begin{pmatrix} 117 & 0 & 0 \\ 0 & 21 & 5\\ 0 & 17& 8\\ \end{pmatrix} }\limits ^{\text{ True } \text{ label }}} \quad CM^{Table\,2}_{optimized}= {\mathop { \begin{pmatrix} 117 & 0 & 0 \\ 0 & 11 & 3\\ 0 & 27& 10\\ \end{pmatrix}}\limits ^{\text{ True } \text{ label }}}$$

**Table 2:** The first dataset’s (Stratified K Fold split) experimental results include a classification report from (a) standard threshold $\lambda = 0.5$, and (b) with threshold moving optimization $\lambda =0.44$.

	Precision	Recall	F1-score	Support		Precision	Recall	F1-score	Support
	No threshold					Threshold moving			
Normal	1.00	1.00	1.00	117	Normal	1.00	1.00	1.00	117
Suspicious	0.81	0.55	0.66	38	Suspicious	0.79	0.29	0.42	38
Pathological	*0.32*	** 0.62**	*0.42*	13	Pathological	0.27	** 0.77**	0.40	13
Accuracy	–	–	0.87	168	Accuracy	–	–	0.82	168
Mmacro avg	0.71	0.72	0.69	168	Macro avg	0.69	0.69	0.61	168
Weighted avg	0.90	0.87	0.88	168	Weighted avg	0.90	0.82	0.82	168

Significant values are in bold and italics.

**Table 3:** Optimization results on function score ($f_{score}$) and corresponds to the each threshold ($\lambda$), refer Line 12 in Alg 1.

	$\lambda$=0.44	$\lambda$=0.40	$\lambda$=0.36	$\lambda$=0.32	$\lambda$=0.28	$\lambda$=0.24
$f_{score}$	**1.35**	** 1.35**	**1.35**	1.30	1.30	1.30

	$\lambda$=0.20	$\lambda$=0.16	$\lambda$=0.12	$\lambda$=0.08	$\lambda$=0.04	$\lambda$=0.00
$f_{score}$	1.25	1.25	1.25	1.25	1.25	1.16

Significant values are in bold.

**Table 4:** The second dataset’s (Stratified K Fold split) experimental results include a classification report from (a) standard threshold $\lambda = 0.5$, and (b) with threshold moving optimization $\lambda = 0.44$.

	Precision	Recall	F1-score	Support		Precision	Recall	F1-score	Support
	No threshold					Threshold moving			
Normal	1.00	1.00	1.00	117	Normal	1.00	1.00	1.00	117
Suspicious	0.76	0.50	0.60	38	Suspicious	0.79	0.29	0.42	38
Pathological	*0.24*	*0.50*	*0.32*	12	Pathological	**0.25**	**0.75**	** 0.38**	12
Accuracy	–	–	0.85	167	Accuracy	–	–	0.82	167
Macro avg	0.67	0.67	0.64	167	Macro avg	0.68	0.68	0.60	167
Weighted avg	0.89	0.85	0.86	167	Weighted avg	0.90	0.82	0.82	167

Significant values are in bold and italics.

$$CM^{Table\,4}_{default}= \begin{pmatrix} 117 & 0 & 0 \\ 0 & 19 & 6\\ 0 & 19& 6\\ \end{pmatrix} \quad CM^{Table\,4}_{optimized}= \begin{pmatrix} 117 & 0 & 0 \\ 0 & 11 & 3\\ 0 & 27& 9 \\ \end{pmatrix}$$

**Table 5:** The third dataset’s (Stratified K-Fold split) experimental results include a classification report from (a) standard threshold $\lambda = 0.5$, and (b) with threshold moving optimization $\lambda = 0.44.$.

	Precision	Recall	F1-score	Support		Precision	Recall	F1-score	Support
	No threshold					Threshold moving			
Normal	1.00	1.00	1.00	117	Normal	1.00	1.00	1.00	117
Suspicious	0.64	0.47	0.55	38	Suspicious	0.50	0.18	0.27	38
Pathological	*0.09*	*0.17*	*0.12*	12	Pathological	**0.14**	**0.42**	**0.21**	12
Accuracy	–	–	0.82	167	Accuracy	–	–	0.77	167
Macro avg	0.58	0.55	0.55	167	Macro avg	0.55	0.53	0.49	167
Weighted avg	0.85	0.82	0.83	167	Weighted avg	0.82	0.77	0.78	167

Significant values are in bold and italics.

$$CM^{Table\,5}_{default}= \begin{pmatrix} 117 & 0 & 0 \\ 0 & 10 & 6\\ 0 & 28& 5\\ \end{pmatrix} \quad CM^{Table\,5}_{optimized}= \begin{pmatrix} 177 & 0 & 0 \\ 0 & 5 & 3\\ 0 & 33 & 8\\ \end{pmatrix}$$

### Comparison between the proposed model with the baseline

**Figure 1:**
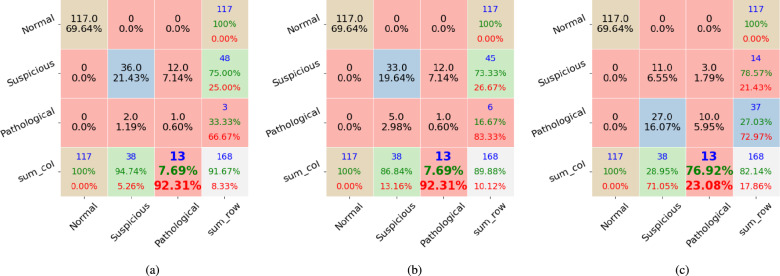
The first dataset’s experimental results include confusion matrix outputs from (**a**) the RF classifier, (**b**) the PRF classifier, and (**c**) the proposed method. Each column represents instances of one class. The total ‘Normal,’ ‘Suspicious,’ and ‘Pathological’ cases are 117, 38, and 13, respectively. The RF and PRF incorrectly classified 12 out of 13 ‘Pathological’ cases. The proposed method has a 76.92% accuracy on the ‘Pathological’ case.

**Figure 2:**
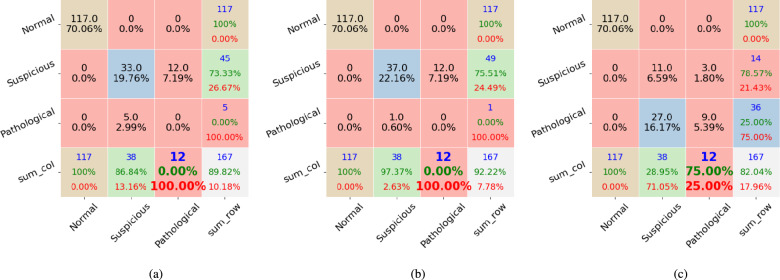
The second dataset’s experimental results include confusion matrix outputs from (**a**) the RF classifier, (**b**) the PRF classifier, and (**c**) the proposed method. Each column represents instances of one class. The RF and PRF incorrectly classified all 12 ‘Pathological’ cases. The proposed method has a 75% accuracy on the ‘Pathological’ case.

**Figure 3:**
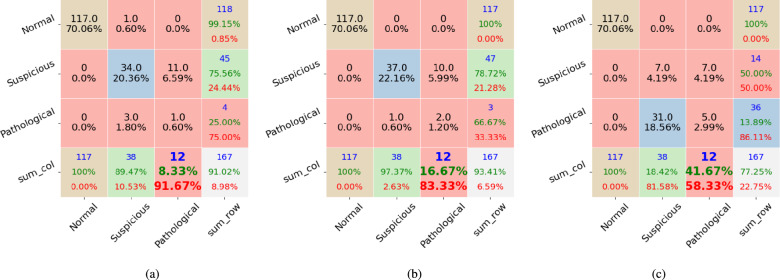
The experiments result on the third data split. Confusion matrix results from (**a**) the RF classifier, (**b**) the PRF classifier, and (**c**) the proposed method. The ‘Normal,’ ‘Suspicious,’ and ‘Pathological’ cases are 117, 38, and 12, respectively. In terms of the ‘Pathological’ case, The RF and PRF correctly classified one case, whereas the proposed method correctly classified 5 cases.

We applied the proposed method to the public dataset CTU-UHB, Employing different feature extraction techniques with varying numbers of features. Therefore, comparing our results with those of other algorithms is unfair. However, we provide a table that lists recent publication articles and their classification results as a reference. Meanwhile, we experimented with baseline classifiers RF and PRF, comparing their results with those obtained by our proposed method. All the compared algorithms were applied to the same experimental environments with 25 estimators, and the max depth per tree is 10. The results are illustrated in Figs. 1, 2, and 3.

Figure 1 shows the classification results of RF (see Fig. 1a), PRF (see Fig. 1b), and our proposed method (see Fig. 1c). The proposed method achieves a higher accuracy in labeling ‘Pathological’ cases, correctly classifying 9 cases compared to 0 by RF and 2 by PRF. Moreover, the proposed method misclassified 2 cases of ‘Pathological’ as ‘Suspicious’, with 9 errors less than RF and 7 errors less than PRF. Although PF and PRF successfully classify 36 ‘Suspicious’ cases, they misclassify ‘Pathological’ cases as ‘Suspicious’, potentially causing serious problems without garnering the necessary attention. In contrast, the proposed method misclassifies ‘Suspicious’ cases as ‘Pathological’, enabling doctors to choose proper care with or without further treatment based on careful examination.

Figure 2 shows the results of the second data split test by RF in Fig. 2a, PRF in Fig. 2b, and the proposed method in Fig. 2c. The proposed method has the lowest number of misclassified ‘Pathological’ cases of value 6, and RF and PRF misclassify the ‘Pathological’ cases as ‘Suspicious’ with 11 and 10 instances, respectively. All three methods successfully classified all ‘Normal’ cases. The proposed method classified 20 instances incorrectly from ‘Suspicious’ to ‘Pathological’, whereas RF and PRF misclassified three and two cases, respectively. PRF successfully classifies one ‘Pathological’ case, RF failed to recognize any ‘Pathological’ case, and the proposed method correctly classifies five cases. Figure 3 shows that the proposed method outperforms PRF and RF methods on the ‘Pathological’ classification tasks, and the proposed method got three misclassified cases on ‘Pathological’. Meanwhile, RF and PRF misclassified ‘Pathological’ to ‘Suspicious’ with 10 cases for each. Overall, the proposed method improves the accuracy of pathological case classification by combining multiple fusion techniques.

### Sampling method selection and baseline classifier

We selected the RandomUndersampling technique to address the problem of imbalanced data. In this section, we conducted experiments to evaluate different sampling techniques and their results, empirically presenting that undersampling is good for minority-class data samples, especially for our CTG data. In addition, we illustrate the benefits of the selection of the baseline classifier.

#### Selection on sampling method

We selected four oversampling methods^24^: *SMOTE*^25^, *ADASYN, Borderline-SMOTE (Border), RandomOverSampler (RandOver)*, and four undersampling methods^26^: *RandomUnderSampler (RandUnder), RepeatedEditedNearestNeighbours (RENN), OneSidedSelection, TomekLinks*.

We applied each sampling method before fitting the data into a Random Forest classifier and compared the overall accuracy of all cases and recall score on the Pathological class data. Each experiment was carried out five times and Tables 6 and 7 show the general average precision and recall scores (based on pathological data).

**Table 6:** The average of overall accuracy $\frac{1}{5}\sum _{i=1}^5$*acc*$\phantom{0}_i$ by each sampling method ran five times.

	Oversampling				Undersampling			
Method	SMOTE	ADASYN	Border	RandOver	RandUnder	RENN	OneSided	TomekLinks
	0.894	0.910	0.916	0.902	0.843	0.773	0.892	0.888

**Table 7:** The average Recall score for Pathological cases by each sampling method ran five times.

	Oversampling				Undersampling			
Method	SMOTE	ADASYN	Border	RandOver	RandUnder	RENN	OneSided	TomekLinks
	0.079	0.211	0.286	0.075	0.396	0.975	0.214	0.296

Table 6 shows the average precision in all cases. Generally, the oversampling method performs better than the undersampling methods, where Borderline-SMOTE achieved 0.916 average accuracy on all from the Oversampling group, and the highest score for the Undersampling score is achieved by TomekLinks with 0.888. In addition, the lowest score for the sampling group is 0.894, which is still higher than the one obtained by TomekLinks.

It should be noted that we are more interested in pathological cases than in other cases. In other words, we pay more attention to the metric score on the Pathological cases. Table 7 shows the average recall score in pathological cases. From Table 7, we observe that the subsampling methods perform better than the oversampling methods in general cases. The lowest recall score for the Undersampling method is 0.214, which is better than the majority methods from the Oversampling group. Surprisingly, we found that the RepeatedEditedNearestNeighbors (RENN) method can achieve a recall score of 0.975, and the RandomUndersampling method ranks second with a recall score of 0.396. In this study, we choose RandomUndersampling for the sampling method, which does not require an additional parameter requirement, whereas RepeatedEditedNearestNeighbours needs to assign neighbor size, which influences the results.

Table 6 shows the average precision in all cases. Generally, the oversampling methods perform better than the undersampling methods. Borderline-SMOTE achieved an average accuracy of 0.916, the highest among oversampling methods, while TomekLinks achieved the highest score among undersampling methods with 0.888. In particular, the lowest score for the oversampling group was 0.894, which is still higher than the score obtained by TomekLinks.

It is important to note that we are more interested in pathological cases than other cases. In other words, we focus more on the metric scores for the pathological cases. Table 7 shows the average recall score for pathological cases. From Table 7, we observe that the undersampling methods generally perform better than the oversampling methods in these cases. The lowest recall score for the undersampling methods is 0.214, which is better than the majority of methods in the oversampling group. Surprisingly, we found that the RepeatedEditedNearestNeighbours (RENN) method can achieve a recall score of 0.975, and the RandomUndersampling method ranks second with a recall score of 0.396. In this study, we chose RandomUndersampling as the sampling method since it does not require additional parameter tuning, whereas RepeatedEditedNearestNeighbours needs to specify the neighbor size, which influences the results.

#### Selection on classifier

In our implementation, we selected the Random Forest and Probabilistic Random Forest classifiers for the following reasons: *1)* Out-of-Bag (Oob) Estimation: eXtreme Gradient Boosting (XGBoost)^27^ does not natively support Out-of-Bag (Oob) estimates, although we can simulate this by evaluating the model on the training dataset or a separate validation dataset. Accurate OOB estimates are crucial, as they can influence the optimization of the probability threshold. Thus, we prefer the Random Forest classifier, which inherently provides OOB scores. *2* Fuzzy Data Splits: The Probabilistic Random Forest classifier simulates data splits using a fuzzy concept. This approach is particularly suitable for the CTG dataset and enhances the classifier’s performance.

### Feature importance and explainability

To understand the contribution of individual features to the CTG classification performance, we analyzed the importance weights learned by the ensemble classifier. Therefore, we applied the ELI5 https://pypi.org/project/eli5/ tool to the trained Random Forest model to predict testing data using the importance of permutation-based features. The extracted features include time-domain descriptors, frequency-domain attributes, autocorrelation-based statistics, EMG-inspired features (Jx-EMGT)^28,29^, and clinically relevant morphological characteristics such as baseline, decelerations, and accelerations^30,31^. These categories capture a range of signal properties from general variability and spectral energy to transient events associated with fetal stress responses.

**Figure 4:**
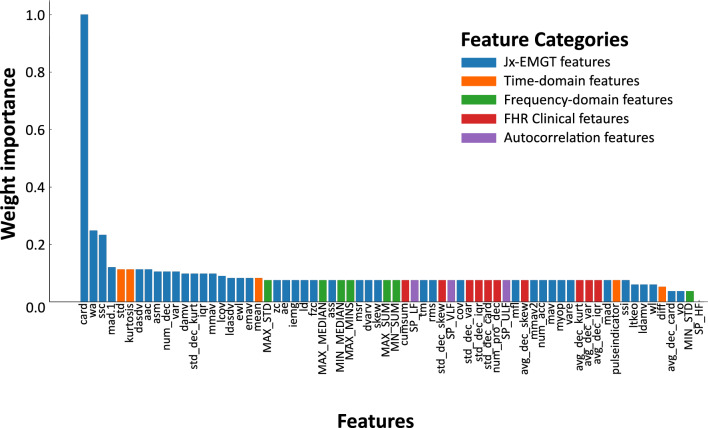
Ranked importance of all extracted features used in CTG classification. Feature bars are color-coded by their category, including time-domain (orange), frequency-domain (green), autocorrelation (purple), Jx-EMGT or EMG-inspired (blue), and clinical fetal heart rate morphological features (red). The dominance of EMG and time-domain metrics is evident among the highest-ranked inputs. Please refer to Table 15 for feature name abbreviations and their full terms.

The ranked feature importance (see Fig. 4) revealed a strong dominance of Jx-EMGT and time-domain features, which together made up 8 of the 10 most important predictors. This reflects their ability to capture the variability and complexity of FHR dynamics, which are key markers of fetal well-being. It is likely that the strong class imbalance in the dataset—favoring normal cases—also biases the model toward features that effectively describe *physiological* FHR patterns, such as baseline variability and acceleratory behavior. These patterns tend to be more consistent and abundant in normal recordings, making them statistically dominant in the learning process.

The single most important feature was *cardinality* (card), a Jx-EMGT metric that quantifies the number of unique values in the signal segment. This measure has also been shown to be highly relevant in EMG-based classification tasks, where it reflects neuromuscular responsiveness^32^. In the CTG context, higher cardinality likely correlates with complex and adaptive heart rate behavior—an indicator of autonomic reactivity and fetal vitality. Its top ranking thus supports the physiological relevance of signal complexity as a potential digital biomarker.

Although less dominant overall, certain morphological features related to pathological states did appear among the more important variables. Notably, the *number of decelerations* (num_dec) was moderately ranked. This is clinically intuitive, as repetitive or prolonged decelerations are associated with fetal hypoxia and acidosis. Their lower relative importance here is likely due to their infrequent occurrence in the dataset rather than a lack of diagnostic value. Future work with more balanced datasets may better capture their true discriminative power.

**Figure 5:**
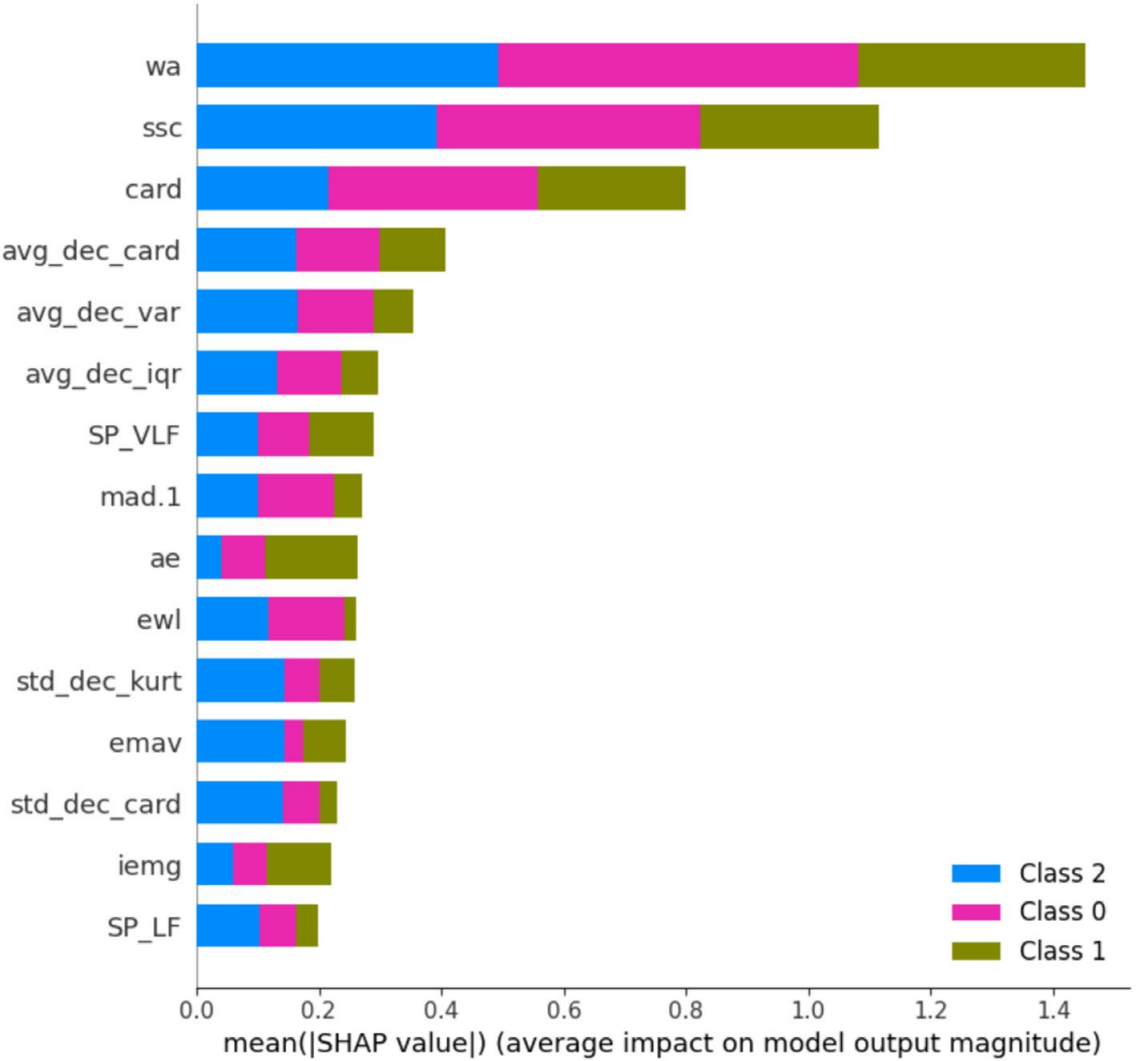
SHAP feature importance for three classes (0: Normal, 1: Suspicious, 2: Pathological). Please refer to Table 15 for feature name abbreviations and their full terms.

Based on the SHAP value analysis as shown in Fig. 5, the feature importance plot highlights the critical role of the metrics related to deceleration in determining pathological cases (Class 2) within the processed CTG dataset, where Class 0, 1, and 2 represent Normal, Suspicious, and Pathological conditions, respectively. Features such as ‘num_dec‘ (number of decelerations), ‘avg_dec_card‘ (average cardinality value during decelerations), and ‘std_dec_kurt‘ (standard deviation kurtosis during decelerations) exhibit significant SHAP values, indicating their substantial influence on the model’s decision to classify instances as Pathological. This underscores the importance of deceleration patterns, which reflect abnormal fetal heart rate reductions, as key indicators to identify high-risk cases, aligned with clinical priorities in maternal and fetal health monitoring. Other features like ‘mean‘ (average value) and ‘std‘ (standard deviation) of the signals also contribute, but decelerations stand out as pivotal in distinguishing Pathological outcomes from Normal and Suspicious cases.

It should be noted that during the SHAP explanation, we adjust the predict proba function that integrates the moving threshold into the SHAP explanation process, ensuring that the feature importance reflects the final class assignments (Normal, Suspicious, Pathological) in our dataset, rather than just the raw probabilities.

**Table 8:** Overview of AI-based techniques and their use for fetal state classification. Here, Recall(P) and F1(P) indicates the recall and F1 score on Pathological class and ACC is overall accuracy. The proposed algorithm focuses on improving minority class classification. However, direct comparison of results is challenging due to varying group numbers in classification tasks.

**Author, source**	**Method**	**Groups**	**Features**	**Dataset**	**Result**
Georgieva et al. ^33^	ANN	2	12	own	SE = 60.3%
					SP = 67.5%
Tang et al. ^34^	SVM	2	9	own	ACC = 83.46%
	RF				ACC = 84.5%
	RNN				ACC = 90.3%
	CNN				ACC = 94.7%
Zhao et al. ^14^	CWT–CNN	2	N/A	CTU-UHB	ACC = 98.34%
					SE = 98.22%
					SP=94.87%
Zhao et al. ^13^	RP–CNN	2	N/A	CTU-UHB	ACC = 98.69%
					SE = 99.29%
					SP=98.1%
Comert et al. ^20^	ANN	2	12	CTU-UHB	ACC = 77.71%
	k-NN				ACC = 70.47%
	DT				ACC = 79.34%
	SVM				ACC = 88.58%
Jezewski et al. ^18^	FC$\epsilon$H	2	12	CTU-UHB	SE=74.55%
					PPV=40.17%
					SP=90.1%
					NPV=97.63%
Proposed	Probability random forest	**3**	68	CTU-UHB	ACC = 82%
without tuning	Cohen kappa, threshold moving				Recall(P) = 64%
					F1(P) =33%

Significant values are in bold.

### Result comparison with other classifier with hyperparameter tuning

To evaluate the performance of various machine learning models in classifying CTG data, which is critical to assessing fetal health during pregnancy, we compared five methods: RF, PRF, SVM, Logistic Regression, and a proposed ensemble method combining RF and PRF with threshold tuning. As previously, we classify fetal heart rate patterns into three categories: normal, suspicious, and pathological, the pathological class being the most critical to accurately identify due to its implications for maternal and fetal health. Each method was optimized using Optuna, a hyperparameter tuning framework, to maximize the precision of the classification, with particular emphasis on the precision of the pathological class. The tuning process involved a stratified k-fold cross-validation (k = 3) to ensure a robust evaluation, and RandomUnderSampler https://imbalanced-learn.org/stable/ was applied to address the class imbalance, which is common in medical data sets such as CTG.

Hyperparameter tuning was performed over specific ranges for each method, as detailed in the table below. For RF and PRF, we tuned the number of estimators and maximum tree depth, with PRF additionally optimizing ‘keep_proba‘ and ‘new_syn_data_frac‘ to control synthetic data generation. SVM tuning focused on the regularization parameter ‘C‘ and kernel coefficient ‘gamma‘, while Logistic Regression tuned ‘C‘ and the solver type. The ensemble method optimized parameters for both the RF and PRF components, along with a threshold to combine their probability outputs. Table 9 summarizes the parameter ranges used in the Optuna optimization.

**Table 9:** Hyperparameter ranges for compared classifiers’ optimization.

Method	Parameter	Range
Random forest	n_estimators	[15, 25]
	max_depth	[5, 15]
Probability randomForest	n_estimators	[15, 25]
	max_depth	[5, 15]
	keep_proba	[0.3, 0.7]
	new_syn_data_frac	[0.05, 0.3]
SVM	C	[0.01, 1] (log scale)
	gamma	{scale, auto}
Logistic regression	C	[0.01, 1] (log scale)
	solver	{lbfgs, liblinear}
	max_iter	1000 (fixed)
Proposed ensemble	rf_n_estimators	[15, 25]
	rf_max_depth	[5, 15]
	prf_n_estimators	[15, 25]
	prf_max_depth	[5, 15]
	keep_proba	[0.3, 0.7]
	new_syn_data_frac	[0.05, 0.3]
	threshold	[0.0, 0.44], step=0.02

The results in Table 10, derived from our CTG dataset, highlight the superior performance of our proposed ensemble method in identifying the pathological class, which is essential to ensure timely medical intervention during childbirth. The proposed method achieved the highest pathological precision in all folds, with a maximum of 0.7692 in fold 1, compared to 0.2308 for RF and SVM, 0.1538 for PRF and 0.0833 for Logistic Regression. Although RF and PRF excelled in the accuracies of the Normal and Suspicious class accuracies (averaging  0.9123 for Suspicious), their pathological accuracies were significantly lower. SVM struggled across all classes (average pathological accuracy of 0.1047), and Logistic Regression, despite strong Normal and Suspicious performance, had a maximum Pathological accuracy of only 0.0833. The proposed ensemble method, by combining RF and PRF predictions with an optimized threshold, effectively balanced the trade-off between classes, achieving a robust pathological classification accuracy that is critical for CTG analysis. This shows that our method is well suited for this task, offering a significant improvement in detecting the most critical fetal health conditions.

**Table 10:** Class-wise accuracy comparison across classifier and folds.

Method	Fold	Normal	Suspicious	Pathological	Best in pathological
Random forest	Fold 1	1.0000	0.9474	0.2308	0.2308
	Fold 2	1.0000	0.9211	0.0000	0.2308
	Fold 3	1.0000	0.8684	0.0833	0.2308
	Average	1.0000	0.9123	0.1047	0.2308
SVM	Fold 1	0.4701	0.3684	0.2308	0.2308
	Fold 2	0.6581	0.5263	0.0833	0.2308
	Fold 3	1.0000	0.0000	0.0000	0.2308
	Average	0.7094	0.2982	0.1047	0.2308
Logistic regression	Fold 1	1.0000	0.9211	0.0769	0.0833
	Fold 2	1.0000	0.8684	0.0833	0.0833
	Fold 3	1.0000	0.9211	0.0833	0.0833
	Average	1.0000	0.9035	0.0812	0.0833
Probability random forest	Fold 1	1.0000	0.8947	0.1538	0.1538
	Fold 2	1.0000	0.9211	0.0833	0.1538
	Fold 3	1.0000	0.9211	0.0833	0.1538
	Average	1.0000	0.9123	0.1068	0.1538
Proposed method	Fold 1	1.0000	0.2895	0.7692	0.7692
	Fold 2	1.0000	0.1579	0.6667	0.7692
	Fold 3	1.0000	0.2632	0.5000	0.7692
	Average	**1.0000**	0.2369	**0.6453**	**0.7692**

Significant values are in bold and underline.

**Table 11:** Overall statistical test results by STAC platform^35^.

Statistic	*p*-value	Result
2.04494	0.18063	H0 is accepted

Using the STAC web platform^35^, we performed statistical tests to compare the performance of five machine learning classifiers shown in Table 11. The overall statistical test yielded a statistic of 2.04494 with a p-value of 0.18063, accepting the null hypothesis (H0) that there is no significant difference in performance among the algorithms. However, the ranking placed the proposed method ranked highest. As only one data set was used with a three-fold cross-validation, the results of the statistical analysis may be biased, suggesting caution in interpreting the lack of significant differences.

## Discussion

The experimental results demonstrated that the proposed method exhibits promising performance, and the threshold moving optimization improves the classification of ‘Pathological’ instances. As mentioned previously, the literature on experiments using the CTG dataset varies due to differences in feature size, signal segment regulation, and other factors, leading to a lack of consistent standards and implementations. Consequently, we limited our comparison to the baseline classifiers and presented their performance results. However, Table 8 provides a compilation of relevant results from other studies for reference, while Tables 6 and 7 summarize the results of the methods tested in sampling techniques addressing imbalanced data. However, the study has several limitations that will be discussed in detail herein.

One significant limitation of this study, and a common issue in the field of machine learning-based computer-aided diagnostic systems for CTG, is the lack of available datasets. The current CTU-UHB dataset used in this study is unbalanced and contains a majority of normal cases. This imbalance affects the results, as it is evident that the model performs well in distinguishing normal cases, but the precision in identifying suspicious and pathological cases is lower. However, these are often of greater clinical interest. The second significant drawback of the CTU-UHB database is the poor quality of some recordings, particularly those related to uterine contractions. Due to this, only parameters related to fetal heart rate could be obtained and analyzed. The exclusion of uterine activity is a substantial limitation, as the interaction between uterine contractions and fetal heart rate provides several crucial parameters for an accurate diagnosis. This highlights the necessity for future research to focus on collecting and curating larger and more diverse datasets, ensuring a more balanced representation of all possible CTG outcomes.

Moreover, reliance on hard thresholds for labeling signals based on pH values introduces another layer of complexity. These thresholds may not always accurately reflect the true clinical condition, leading to possible mislabeling of cases. Future work could explore more sophisticated labeling techniques that incorporate a broader range of clinical indicators to provide a more nuanced classification framework. In this study, we chose to classify the CTG signals into three categories: *normal*, *suspicious*, and *pathological*. This decision reflects the clinical decision-making process as described in the FIGO 2015 guidelines, which are widely followed by clinicians. However, other researchers have used only two classes—normal and pathological—based on pH levels. The choice of threshold varies, some using a pH of 7.2 and others 7.15, see Table 12.

Although low pH is often associated with acidosis, it is not always true that a fetus with low pH was affected by hypoxia, nor that a fetus with a high pH was not. The timing of pH measurement also plays a crucial role. The pH value may change over time, and it is important to know whether the pH was taken immediately after birth or several minutes later. Unfortunately, this information is missing in the CTU-UHB database. As a result, there are cases in the dataset where the pH was low (below 7), but the Apgar score was high (above 8, where adverse outcome is usually considered as value below 7). The Apgar score, although subjective, is a widely used parameter to assess neonatal outcomes. This discrepancy can lead to misclassification in our model, as a fetus classified as pathological based on pH might actually be healthy according to Apgar scores. There is no information available on the long-term health outcomes of these fetuses, such as whether they developed seizures or not, making it difficult to definitively classify them. Some researchers have used additional conditioning factors alongside pH values, such as the Apgar score or birth weight percentiles ^18^. However, there is no consensus, either clinically or technically, on the criteria to classify a newborn into specific categories. This lack of standardization complicates the development of universally applicable classification models. As the performance of these systems is heavily influenced by how pathological outcomes are defined, there is a clear need for studies to align the community on these definitions as suggested in ^36^.

Despite these limitations, the study presents a promising approach to addressing data imbalance through ensemble classifiers and probability threshold optimization. The implications of our findings for clinical practice are significant. Our system was designed to integrate seamlessly into current methodologies and processes to assist in diagnosis. We chose a 3-class system (normal, suspicious, pathological) to align with the FIGO 2015 guidelines. However, future iterations of this system could vary. We may opt for a simpler 2-class system that shows the probability of each class or a more complex system that provides a detailed decision-making process using various descriptive or visualization tools. Such enhancements would not only aid clinicians in making more informed decisions but also offer transparency in the diagnostic process, potentially increasing trust and adoption of these systems in clinical settings.

To further enhance the accuracy and reliability of fetal monitoring, quantitative parameters should be investigated that evaluate the entire process, including the health state of both the fetus and the mother, as well as the placenta and uterus. This investigation should encompass constant covariates such as age, family history, and comorbidities, and variable ones such as heart rate variability, contraction count, blood pressure, and/or ECG wave morphology. Moreover, understanding how these factors are linked and change in correlation with each other could provide deeper insights, potentially utilizing advanced methods such as graph theory or coupling maps. In medical applications, the interpretability and explainability of models are increasingly important to gain clinician trust and ensure ethical use. We applied a simple tree-based classifier model, which can be easily interpreted using Explainable AI techniques such as LIME, SHAP, and tree surrogates^37^. This will be the focus of our future work.

This paper addresses the challenge of imbalanced data in the classification of fetal signals from the CTG dataset, a critical issue that can lead to misclassification of pathological cases and improper medical care. While previous studies have identified this problem, limited research has focused on effectively mitigating it. To bridge this gap, we propose a low-cost, efficient multiintegration strategy tailored for real-world imbalanced data.

Our approach combines undersampling to address data imbalance, threshold shifting for conservative classification of specific labels, and an ensemble classifier to improve accuracy and noise robustness. Specifically, we integrate Random Forest (RF) and Probabilistic Random Forest (PRF) classifiers with automatic threshold optimization using the Cohen kappa score for decision-making. In particular, the proposed method does not increase the complexity of time and requires minimal additional space, making it computationally efficient.

Experimental results on the CTG dataset from the CTU-UHB database demonstrate that the method significantly improves minority class performance, particularly for ‘pathological’ cases, while maintaining robustness across other labels. This work highlights the potential for generalizing the proposed strategy to broader real-world applications with imbalanced data.

## Methods

**Figure 6:**
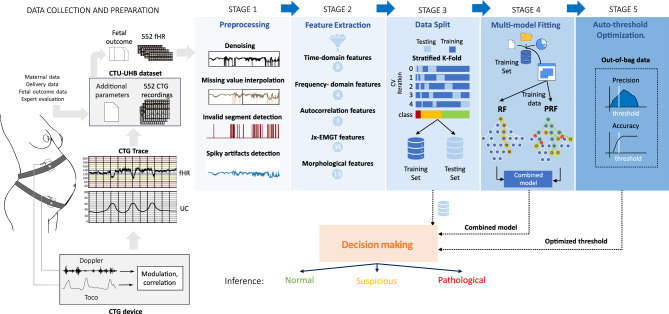
Block diagram of the implementation, including the data collection and preparation and individual stages leading to the decision-making process.

In this section, we introduce the dataset used for the experiments, as well as the detailed implementation of the proposed fetal hypoxia classification system integrated with a probability classifier aided by Cohen’s kappa metric and moving threshold. This model comprises four stages: I) Preprocessing, II) Feature extraction, III) Multi-Model fitting, and IV) Auto-Threshold Optimization. Each stage of the model implementation will be described in the following subsections. A flowchart of the proposed model is shown in Fig. 6; overall, it is composed of four stages: I) Preprocessing, II) Feature extraction, III) Multi-Model Fitting, and IV) Auto-Threshold Optimization. Each stage of the model implementation will be described in the following subsections.

### Data preparation

In this investigation, signal labeling is based on pH values derived from general clinical characteristics. The recordings are classified into three categories: ‘Normal’, ‘Suspicious’, and ‘Pathological’. The pH level indicates the acid-base equilibrium of the blood, offering insight into possible fetal acidosis resulting from intrauterine hypoxia^38^. A pH value greater than 7.20 is classified as the normal class, while pH values between 7.19 and 7.06 fall into the suspicious category. Signals with a pH value below 7.05 are labeled as pathological class. The signal quality is suboptimal, as highlighted in the articles^19,39^. Consequently, we conduct signal segmentation into 10-minute cuts (hereafter denoted as *segments*), and labeling the segmented signal is based on the fetal’s ultimate pH value.

Furthermore, the CTU-UHB dataset initially comprised 552 extracted signal segments. We experimented with 502 segments in this study, and the signal segments were categorized into three class labels: ‘Normal,’ ‘Suspicious,’ and ‘Pathological,’ comprising 351, 114, and 37 instances, respectively. Notably, this dataset exhibits class imbalance. We apply Stratified *K*-Fold splits of the dataset for cross-validation and present the results separately. Ultimately, the test dataset size is around 117, 38, and 13 for ‘Normal,’ ‘Suspicious,’ and ‘Pathological’ for each split.

**Subsequently, we analyze the classification report and consult a doctor about the inconsistencies or controversies between the result and the data.**

### Stage I: Pre-processing

Due to the inadequate quality of the UC signal in the CTG database for analysis, as highlighted in several articles^19,39^, we selectively extract the FHR signal for the study, taking into account both analysis requirements and the available dataset volume. Clinical FHR signals were acquired using a Doppler ultrasound probe placed on the pregnant woman’s abdomen. These signals were vulnerable to various noise sources, including maternal and fetal movements, sensor misplacement, and external environmental factors. Interference noise typically presented as missing values (FHR = 0) and spiky artifacts (FHR > 200 bpm or FHR < 50 bpm).

We employed interpolation techniques to eliminate noise during signal preprocessing^39^, following the specifications below:

- Linear interpolation is utilized for FHR signals with a value of zero but less than 15 seconds in duration; otherwise, they are directly excluded.
- In cases where the FHR signal is unstable (i.e., 25 times/minute difference between adjacent two points), interpolation is performed.
- For FHR values exceeding 200 times/minute or falling below 50 times/minute, we perform Hermite spline interpolation.

**Figure 7:**
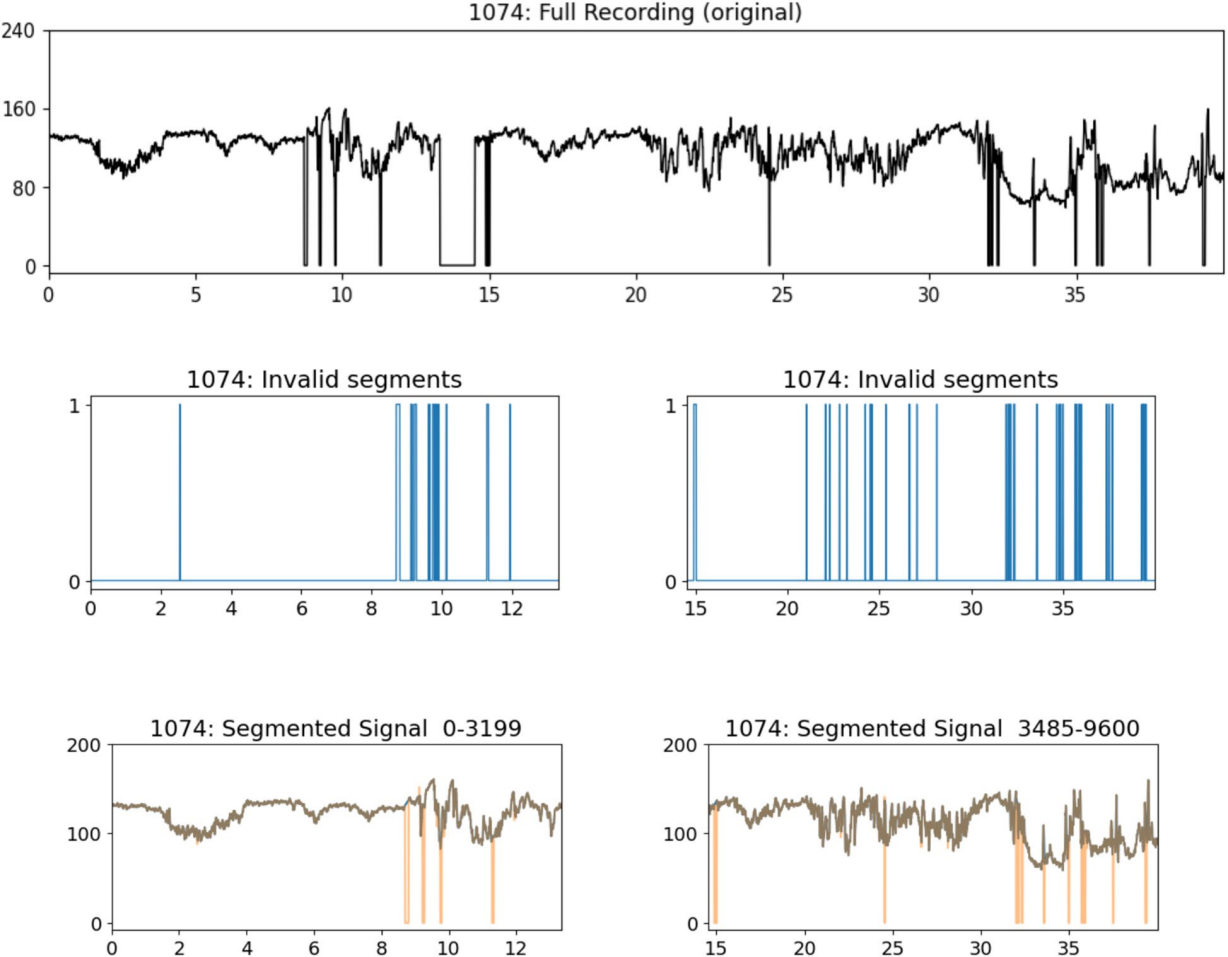
An example of signal segmentation on recording 1074, where the original recording (in black) proceeds with denoising, missing value interpolation, and spiky artifacts detect, produces two segments.

We conduct signal segmentation to ensure uniform signal length for the classifier input, resulting in 10-minute segments. When dealing with signals with a normal label, we prioritize the late valid segment (in time) if multiple choices are available in one recording. In the case of pathological signals, all valid segments are included due to the imbalance in data quantity between pathological and normal recordings. An example of signal segments is shown in Fig. 7.

### Stage II: Feature extraction

Following the generation of 10-minute segments in Stage I, this phase transforms the 1D signal data into tabular feature data.

Feature extraction, also known as feature transformation, facilitates comprehensive qualitative and quantitative interpretation of fetal heart rate (FHR), adhering to standardized guidelines suggested by the National Institute of Child Health and Human Development^31^. In this study, we extract five types of features from FHR: namely time domain features, frequency domain features, autocorrelation features^40^, morphological features, and Jx-EMGT features (specialized for electromyography signal)^28,29^ (Jx-EMGT, source from https://github.com/JingweiToo/EMG-Feature-Extraction-Toolbox). We assume that each specialized feature may carry various information that boosts classification performance.

In summary, the extracted features (we exclude the duplicated ones) include eight time-domain features such as *mean, standard deviation, skew, pulse indicator*, and *kurtosis*; four frequency-domain features related to the lowest, second lowest frequency bin, middle, and highest segments of the spectral energy; seven autocorrelation features from *pyplot-acorr* in the Matplotlib Python package; 36 Jx-EMGT features; and an additional 13 morphological features related to accelerations and decelerations, covering aspects like appearance, interquartile range, skewness, and variance. In particular, the result of the prolonged deceleration detection (a decrease in FHR of > 15 beats per minute measured from the most recently determined baseline rate) is also included, contributing to a total number of 68 features of 502 signal segments.

Measuring morphological features requires the baseline rate information^41^. The baseline FHR is the average heart rate of a fetus during periods of relative stability in a 10-minute segment. It serves as a crucial indicator of fetal well-being and is important in assessing the general health of the unborn baby. Upon baseline, it provides a reference point for understanding accelerations and decelerations. Analyzing the relationship between the baseline and these variations helps healthcare professionals assess the overall fetal condition, ultimately contributing to better pregnancy management and outcomes. In this study, we extract the baseline from the FHR signal by FHRMA^30^ source from https://github.com/utsb-fmm/FHRMA. The detailed name of the feature, the abbreviations and the calculation are shown in Tables 15 and 16, we will release the whole source code via link https://github.com/lingping-fuzzy.

**Table 12:** A description of feature extraction details.

Features	Introduction	Variety	Methods
Time-domain features	*mean, diff, skew, kurtosis, cumsum, etc*	8	Pandas (Python)
Frequency-domain features	Frequency bin, spectral energy	4	NumPy (fast fourier transform) (Python)
Autocorrelation features	Autocorrelation with 240 lags (1mins)	7	Matplotlib (autocorrelation) (Python)
Jx-EMGT features	Electro signal basic feature	36	Jx-EMGT^28,29^
morphological features	Accelerations and decelerations, etc	13	FHRMA (MatLab)^30,30^

### Stage III: Data split

The total number of 502 available signal segments is not quite large for the standard ML classifier. Therefore, we apply a stratified K-Fold cross-validator^42^ to verify the performance of the proposed model. This cross-validation object is a variation of KFold that returns stratified folds. The folds are made by preserving the percentage of samples for each class, which means that it may produce a different number of samples in each split. A summary of data description is shown in Table 13.

**Table 13:** CTU-UHB data description and summary of processed data.

Data	Number of samples	Type	Number of features	Number of classes	Samples per class
Source	552	Signal	–	–	–
Processed	502	Feature numeric value	68	3	(351, 114, 37)

**Figure 8:**
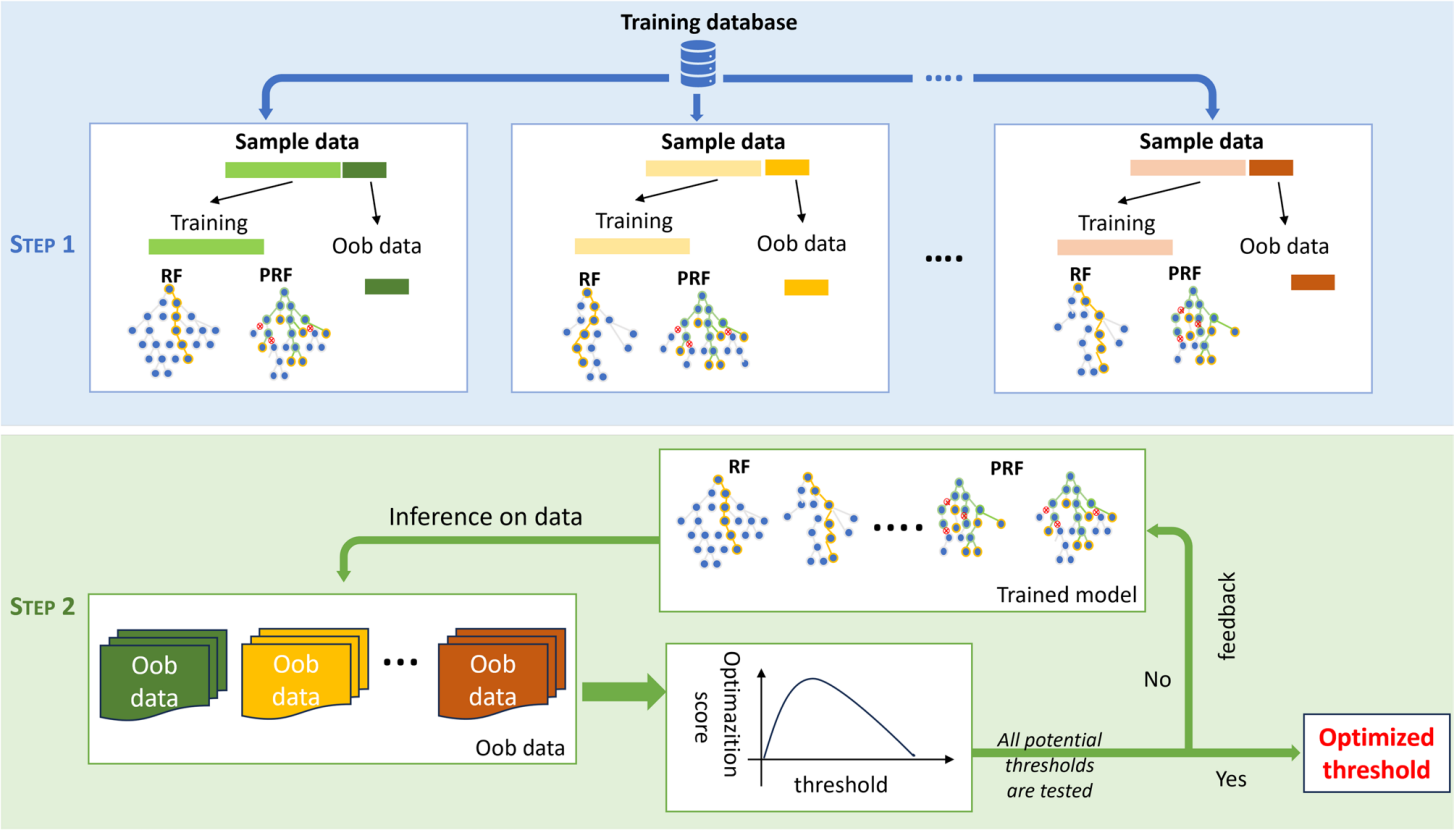
Auto-threshold optimization diagram, where the inference data are from Oob (Out of bags). The optimization step does not increase computation much.

### Stage IV: Multi-model fitting

In this stage, we process the tabular feature data, fit them into two classifiers, analyze the probability distribution, and then make decisions based on the threshold and the achieved probability. As the medical signal classification task is tough, we choose to hybridize the PRF algorithm to boost the performance; PRF suits noisy datasets due to the consideration of vagueness in features and labels rather than fixed deterministic quantities.

First, we apply RF and PRF^23^ separately to train the classifier on the training data, which is undersampled using the RandomUnderSampler method (see Alg 1, lines 4-6). Furthermore, we conducted experiments to evaluate the undersampling and oversampling techniques to select the optimal sampling method; results are shown in Table 6. We extract the corresponding probability results for the Out-of-Bag (Oob) data (lines 7-8). The Oob data refer to the data not used for training in the current fitting state. The prediction probability for the Oob data is saved and used for threshold tuning, avoiding extra training computation.

Next, we combine the probabilities obtained from RF and PRF in the test data by weighted addition, denoted $\textbf{P} = {p_1, p_2, \dots , p_c}$ for each class (lines 7-9). This combined probability will be used to determine the signal’s status on the basis of the optimized threshold.

The best threshold is found through an auto-threshold tuning process, where we define an optimization function (line 12) that calculates a score for each threshold application (details are provided in the next subsection). The value corresponding to the highest function score is considered the best threshold (lines 11-17). Finally, we decide the label of each test signal segment based on the best threshold and the hybrid output probability $\textbf{P}$ (line 18) and generate the report (line 19).

### Stage V: Auto-threshold optimization

This stage addresses the decision-making problem posed by our highly unbalanced CTG signal data, where the number of signals with normal labels far exceeds those with suspicious and pathological ones. Several balanced accuracy metrics are available to assess model performance on imbalanced data; in this study, we opt for Cohen’s kappa, particularly the weighted Cohen’s kappa, in our optimization score function. The flowchart of this stage is shown in Fig. 8.

Auto-threshold tuning iteratively adjusts validation results to find the maximum optimization function score, where we combine Cohen’s kappa score and the precision score on ‘Pathological’ classification, see Alg 1 line [12-13]. To reduce computation costs and training time, we use the prediction probabilities of the Oob data. Algorithm 1 details the auto-threshold optimization process, with notation descriptions in Table 14.

**Table 14:** The notion and description used in Alg 1.

Notion	Description
RF, PRF	Random forest (RF), probabilistic random forest (PRF).
*Oob*	Out-of-bag (Oob), the out-of-bag set is all data not chosen in the sampling process.
*prob*	Probability (prob), RF-prob, the probability produced by RF.
*Oob-probs*	The classification probability on *Oob* data. *RF-Oob-probs* is the probability generated by RF.
$\lambda$	The best probability threshold tuned by the optimization process.
RandomUnderSampler	Synthetic minority undersampling Technique.

We conducted experiments using 502 segments with cross-validation $t=3$, which yielded 3 individual classification reports. To address the bias that arises from the unbalanced data, the synthetic minority RandomUnderSampler technique is applied to the minority label of the data points, as shown in Line 4. Subsequently, we train the RF and PRF separately, and the final prediction probability of test data is derived from the probability combination. Following this, auto-threshold optimization is performed to select the best threshold $\lambda$ based on the optimization function score, which dictates the adjustment of the statistical probability threshold.

**Algorithm 1:**
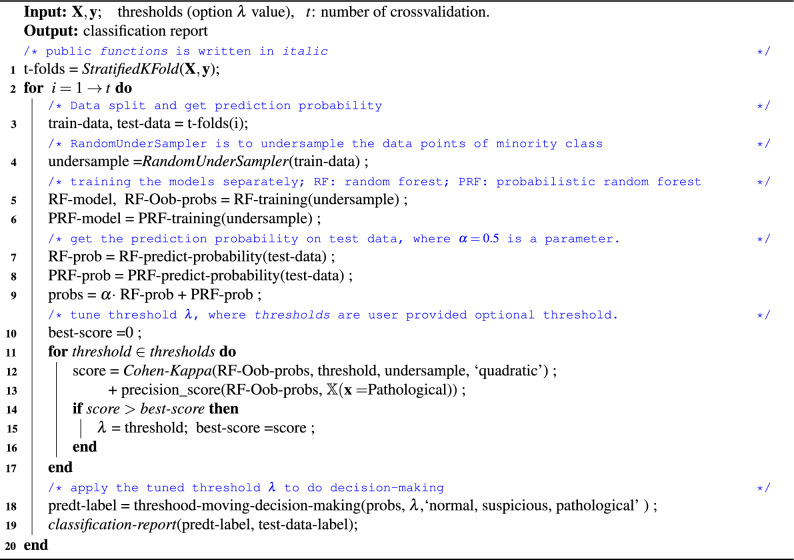
Auto-threshold optimization process

Machine learning classifiers trained on class-imbalanced data are prone to overpredict the majority class, resulting in a higher misclassification rate for the minority class, which often represents the class of interest in many real-world applications. In this work, we propose integrating undersampling, multi-model fitting, and threshold adjustment to address this issue. Undersampling is accomplished using RandomUnderSampler for majority classes of ‘Normal’ cases by randomly picking samples with or without replacement. The multi-model fitting step adapts RF and PRF to balance the classification probability over the noise, while the moving threshold strategy assists in making accurate statistical decisions.

### Classification strategy based on $\lambda$

We optimize the classification threshold for the labels ‘Suspicious’ and ‘Pathological’. This adjustment entails changing the probability threshold for selecting ‘Suspicious’ and ‘Pathological’, while retaining the probability threshold for the ‘Normal’ case. For example, suppose that the final classification probabilities are [0.5, 0.3, 0.2] for ‘Normal’:0.5 ‘Suspicious’,: 0.3 and ‘Pathological’: 0.2; we classify this signal as ‘Normal’; 0.5 is the largest probability value. However, if the probabilities get [0.3, 0.4, 0.5], the case will be categorized as either ‘Suspicious’ or ‘Pathological’ based on the threshold $\lambda$. If ($P(x=$suspicious) - $P(x=$ pathological)) $>\lambda$, the label is ‘Suspicious’; otherwise, we classify it as ‘Pathological’. In the experiment, we propose automatic adjustment of the threshold $\lambda$ based on Oob data and setting the potential thresholds in the range of 0.0 to 0.45.

**Table 15:** Feature abbreviations and names including calculation source code for CTG dataset.

Abbreviation	Name
Time-domain features	
mean	Mean (average value)
std	Standard deviation (measure of spread)
mad	Median absolute deviation (robust variability)
diff	Sum of differences (trend indicator)
skew	Skewness (measure of distribution asymmetry)
pulseindicator	Pulse indicator (max/mean ratio)
kurtosis	Kurtosis (tailedness of distribution)
Frequency-domain features	
MAX_MINS	Total number of local maxima and minima
MIN_SUM	High frequency spectral energy
MAX_SUM	High frequency spectral energy
MIN_MEDIAN	High frequency spectral energy
MAX_MEDIAN	High frequency spectral energy
MIN_STD	High frequency spectral energy
MAX_STD	High frequency spectral energy
Autocorrelation features	
SP_ULF	High frequency spectral energy
SP_VLF	High frequency spectral energy
SP_LF	High frequency spectral energy
SP_HF	High frequency spectral energy
Jx-EMGT features	
emav	Enhanced mean absolute value
ewl	Enhanced wavelength
fzc	New zero crossing
asm	Absolute value of summation of exp root
ass	Absolute value of summation of square root
msr	Mean value of square root
ltkeo	Log teager kaiser energy operator
lcov	Log coefficient of variation
card	Cardinality
ldasdv	Log difference absolute standard deviation
ldamv	Log difference absolute mean value
dvarv	Difference variance value
vo	V-order
tm	Temporal moment
damv	Difference absolute mean value
ar	Auto-regressive model
mad.1	Mean absolute deviation
iqr	Interquartile range
skew	Skewness
kurt	Kurtosis
cov	Coefficient of variation
sd	Standard deviation
var	Variance
ae	Average energy

**Table 16:** Continue-Feature abbreviations and names, including calculation source code for CTG dataset.

Abbreviation	Name
*Jx-EMGT features*	
iemg	Integrated EMG
mav	Mean absolute value
ssc	Slope sign change
zc	Zero crossing
wl	Waveform length
rms	Root mean square
aac	Average amplitude change
dasdv	Difference absolute standard deviation value
ld	Log detector
mmav	Modified mean absolute value
mmav2	Modified mean absolute value 2
myop	Myopulse percentage rate
ssi	Simple square integral
vare	Variance of EMG
wa	Willison amplitude
mfl	Maximum fractal length
num_acc	Number of acceleration
num_dec	Number of deceleration
avg_dec_card	Average cardinality value in signal during deceleration
std_dec_kurt	Standard deviation kurtosis value in signal during deceleration
*Others*	
acc; dec, avg_acc, $\cdots$	Accelerations and decelerations, etc by FHRMA

### Time and space complexity

In this work, we adapt the random forest for its efficient time complexity compared to neural networks. The training process requires $\mathcal {O}(M N^2\log (N))$, where $M$ is the number of trees and $N$ is the number of training samples, with a space complexity of $\mathcal {O}(N\ell )$ to store probability results for the classification labels $\ell$. Notably, the out-of-bag (OOB) results are obtained without additional computation, and the Cohen’s kappa decision does not involve iterative steps. In general, the proposed method maintains the standard time complexity of random forests, with a negligible increase in the complexity of space.

### Feature name and abbreviations

Tables 15 and 16 present the abbreviations and names of the characteristics, including the source, definition, and calculation platform for the metrics used in the CTG dataset.

## Conclusion

This paper addresses the challenge of unbalanced data in the classification of fetal signals from the CTG dataset, a critical issue that can lead to misclassification of pathological cases and improper medical care. Although previous studies have identified this problem, limited research has focused on effectively mitigating it. To bridge this gap, we propose a low-cost, efficient multiintegration strategy tailored for real-world imbalanced data.

Our approach combines undersampling to address data imbalance, threshold shifting for conservative classification of specific labels, and an ensemble classifier to improve accuracy and noise robustness. Specifically, we integrate RFand PRF classifiers with automatic threshold optimization using the Cohen kappa score for decision making. In contrast, traditional methods like SVM and Logistic Regression, while effective for balanced datasets, struggle with imbalanced data due to their reliance on fixed decision boundaries and lack of inherent imbalance handling, often requiring additional techniques like oversampling or class weighting that increase computational overhead. However, the proposed method does not increase the complexity of time and requires minimal additional space, making it computationally efficient.

Experimental results on the CTG dataset from the CTU-UHB database demonstrate that the method significantly improves minority class performance, particularly for ’pathological’ cases, while maintaining robustness across other labels. Compared to SVM and Logistic Regression, which showed lower sensitivity to pathological cases (e.g. due to misclassification of decelerations), the ensemble approach with threshold optimization achieved superior recall and precision, as validated by SHAP analysis that highlighted deceleration metrics. This work highlights the potential for generalizing the proposed strategy to broader real-world applications with imbalanced data, offering a practical alternative to traditional classifiers.

## Data Availability

No datasets were generated or analysed during the current study.
